# Protein Folding Absent Selection

**DOI:** 10.3390/genes2030608

**Published:** 2011-08-16

**Authors:** Thomas H. LaBean, Tauseef R. Butt, Stuart A. Kauffman, Erik A. Schultes

**Affiliations:** 1 Sequenomics LLC, 1428 Chanterelle Lane, Hillsborough, NC 27278, USA;E-Mail: schultes@sequenomics.com; 2 LifeSensors Inc., 271 Great Valley Parkway, Suite 100, Malvern, PA 19355, USA;E-Mail: butt@lifesensors.com; 3 Complex Systems Center University of Vermont, 200C Farrell Hall, 210 Colchester Ave.,Burlington, VT 05405, USA; E-Mail: stuart.kauffman@uvm.edu

**Keywords:** protein folding, evolution, sequence space

## Abstract

Biological proteins are known to fold into specific 3D conformations. However, the fundamental question has remained: Do they fold because they are biological, and evolution has selected sequences which fold? Or is folding a common trait, widespread throughout sequence space? To address this question arbitrary, unevolved, random-sequence proteins were examined for structural features found in folded, biological proteins. Libraries of long (71 residue), random-sequence polypeptides, with ensemble amino acid composition near the mean for natural globular proteins, were expressed as cleavable fusions with ubiquitin. The structural properties of both the purified pools and individual isolates were then probed using circular dichroism, fluorescence emission, and fluorescence quenching techniques. Despite this necessarily sparse “sampling” of sequence space, structural properties that define globular biological proteins, namely collapsed conformations, secondary structure, and cooperative unfolding, were found to be prevalent among unevolved sequences. Thus, for polypeptides the size of small proteins, natural selection is not necessary to account for the compact and cooperative folded states observed in nature.

## 1. Introduction

Fifty years ago, it was observed that proteins fold to complex, aperiodic structures. This observation created a conundrum: since a molecule of such high molecular mass was expected to be overwhelmed by conformational entropy, it was therefore expected that protein folds would resemble amorphous materials such as liquids or glasses, rather than the observed native states, more reminiscent of organic crystals [1]. Although there was at the time no physical theory accounting for how proteins reached stable and specific folds, Darwin's theory of natural selection did explain why proteins folded—so as to acquire specific biochemical functions. This fusion of structural biology and Darwinian theory implied that unevolved sequences (*i.e.*, random-sequence proteins) would rarely have biological function and would likely be disordered [2,3].

Over time however, a number of observational [4–7], experimental [8] and theoretical [9–11] results have contradicted these early assumptions and it now appears that unique, compact protein folds may be much more common throughout sequence space than once presumed. For example, structural studies of natural and engineered mutant proteins have revealed globular, biological proteins to be extremely tolerant of amino acid substitutions [12], deletions [13], insertions including segmental substitutions [14] and circular permutations [15]. Studies comparing structurally homologous proteins from different species demonstrated that a mere 5–20% of a given protein's amino acid sequence remains invariant during evolution, and thus, point mutations appear to be tolerated at the majority of positions. Indeed, it has been demonstrated that sequences with little if any measurable sequence identity can nonetheless fold to identical 3D structures [16,17]. These observations imply, and subsequent theoretical studies have predicted, that any native fold is associated with a set of sequences, often connected by single amino acid mutations. These interconnected sets of sequences are referred to as neutral networks [18]. Distinct neutral networks are presumably interwoven in sequence space and might in some cases exist in close proximity to one another [19,20]. Rather than being statistical anomalies, native folds may be ubiquitous. Indeed, native folds associated with extensive neutral networks are said to have high “designability” [21].

These insights into the relationship between sequence, structure and natural selection are circumstantial however, confounded by the genealogical relationships inherent to biological data sets. Perhaps this mutational robustness is itself a derived, evolutionary adaptation? To acquire information about protein folding unbiased by evolution, it is necessary to perform structural characterizations on sequences that are explicitly outside biological and engineered data sets. Such synthetic, random-sequence polypeptides would have no sequence information relationship to evolved proteins nor to each other. Any physiochemical properties associated with these random-sequence polypeptides would therefore be independent of natural selection and historically contingent constraints of genealogical descent.

As the raw material for evolution, the occurrence of native, or even partially folded conformations among random-sequence polypeptides would have profound implications for the diversification and emergence of folds in the course of evolution. Despite the very large size of protein sequence space (20^N^, where N is the length of the sequence), even a relatively small number of such random-sequence polypeptides could potentially test the early assumptions that unevolved proteins must be disordered.

Along these lines, there have been a number of experimental studies that have synthesized and characterized random-sequence polypeptides. The earliest attempts at chemical synthesis of proteins preceded molecular cloning techniques and solid-phase synthesis methods. At that time, amino acid polymers were produced by random co-polymerization of mixed amino acid N-carboxyanhydrides, containing side-chain blocking groups where necessary [22]. Solution synthesis methods lack control over product length, thus a wide range of polymer sizes was present in any particular sample, with a major fraction often far longer than biological proteins. Furthermore, the copolymers contained only a few types of amino acids, leading to unnaturally biased samples of sequence space. Intriguingly, despite these limitations, evidence of solubility and compactness was demonstrated [23,24].

More recently, genetically-encoded combinatorial libraries of arbitrary proteins have been developed, allowing more precise control over the length and composition of the synthetic arbitrary sequences. Davidson and Sauer [25,26] used synthetic DNA templates encoding 70–90 amino acid positions to express in *E. coli*, arbitrary sequences of glutamine (Q), arginine (R) and leucine (L). Although circular dichroism, gel filtration and analytical centrifugation demonstrated evidence for secondary structure including cooperative unfolding, these QRL sequences, presumably because of their extreme degenerate amino acid composition, were unlike biological proteins in being remarkably resistant to chemical and thermal denaturation. QRL polypeptides were also relatively insoluble in the absence of denaturant. Doi *et al.* [27] used synthetic DNA templates encoding 115–144 amino acid positions, using 5 types of amino acids (valine, alanine, asparagine, glutamine, glycine: VANQG). Intriguingly, this limited “primordial” amino acid composition was found to have higher solubility than biological proteins composed of 20 amino acid types with comparable hydrophobicity. However, these VANQG proteins demonstrated less secondary structure than did the QRL sequences, presumably due to the presence of the helix breaker, glycine. Prijambada *et al.* [28] expressed random proteins having all 20 amino acids (95 random positions in a 141 synthetic construct) although they found that 20% of these sequences were soluble, they did not report detailed biophysical characterizations of individual clones. Most recently, Chiarabelli *et al.* [29] used phage display techniques and a tripeptide thrombin tag to isolate 79 random-sequence proteins having 20 amino acids types, with evidence that 20% were likely folding. However, the sequences analyzed in this study were only 50 residues in length, putting them at the extreme low-end for globular structures.

**Figure 1:**
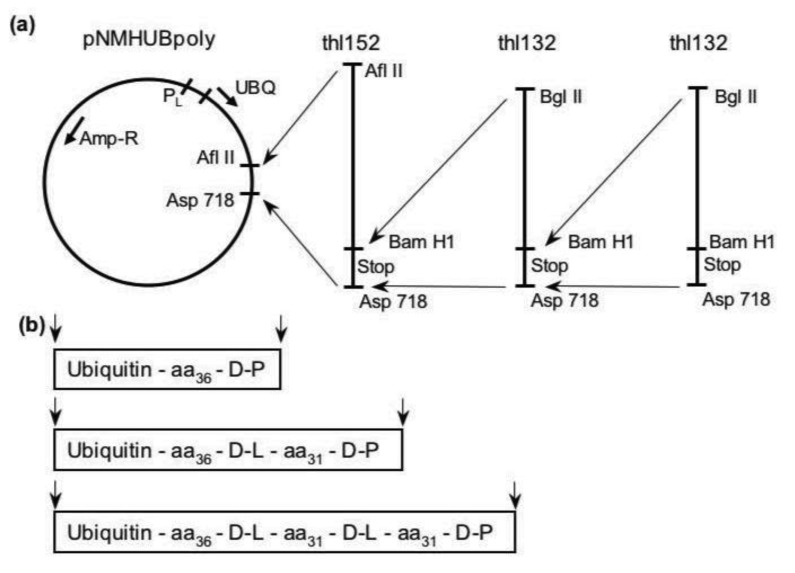
Cloning scheme for production of plasmid libraries encoding random-sequence fusions with ubiquitin. (**a**) The plasmid pNMHUBpoly contains a synthetic gene which encodes human ubiquitin and is controlled by a λ-phage PL promoter. The double-stranded fragments, thl152 and thl132, were produced by restriction digestion of DNA obtained from PCR on the synthetic oligonucleotides described in the text. These inserts were designed with a codon distribution encoding a biologic-like amino acid composition (see details in Section 3.1). Ligation of a Bgl II/Asp 718 digested thl132 fragment with the Bam HI/Asp 718 digested plasmid library results in loss of the original Bam HI site and introduction of a new site downstream, thus additional fragments can be inserted by redigestion of plasmid with Bam HI without internal cleavage of the novel genes; and (**b**) The insertion of a single thl152 fragment into the poly-cloning site following the ubiquitin gene results in 38 amino acid fusions as shown beneath the DNA. Insertion of one or more thl132 fragments results in elimination of an in-frame termination codon, addition of a down-stream stop codon, and elongation of the open reading frame by 33 codons per fragment. The libraries examined herein were LIB38 which contained a single insert and LIB71 which contained two inserts.

The strategy introduced here combines a new method for encoding such polypeptides with an expression system capable of efficiently producing arbitrarily long, compositionally controlled random-sequence polypeptides [30] (see Figure 1 and Experimental Section). The random-sequence proteins were expressed in *E. coli* as carboxy-terminal fusions with ubiquitin via the pNMHUBpoly plasmid [31]. One of the remarkable properties of the expression system is that fused proteins are stabilized by ubiquitin during expression, yet they are able to maintain their own autonomous structure [32]. Expression of proteins as fusions with ubiquitin in this system has been shown to result in high product yield (up to 60 mg fusion per liter culture) using a simple purification procedure [33,34]. The fusions can be processed using specific ubiquitin-carboxy extension hydrolases which faithfully cleave fusions at the C-terminus of ubiquitin [35]. The present approach represents a successful “shotgun” sampling of protein sequence space and lends itself well to the characterization of diverse pools of novel proteins and large numbers of individual sequences. We emphasize that our purpose here was not to probe the detailed structure of individual isolates, but to observe the generic folding properties of proteins sampled randomly from a defined compositional domain of sequence space where biological proteins are known to exist. This is an analysis of sequence space, not molecules.

As such, the exact amino acid sequences or the details of folded conformation of the isolates are not necessary in answering the questions we pose or to the conclusions reported herein.

## 2. Results and Discussion

### 2.1. Demonstration of Secondary Structure in Random-Sequence Fusion Protein Pools

**Figure 2:**
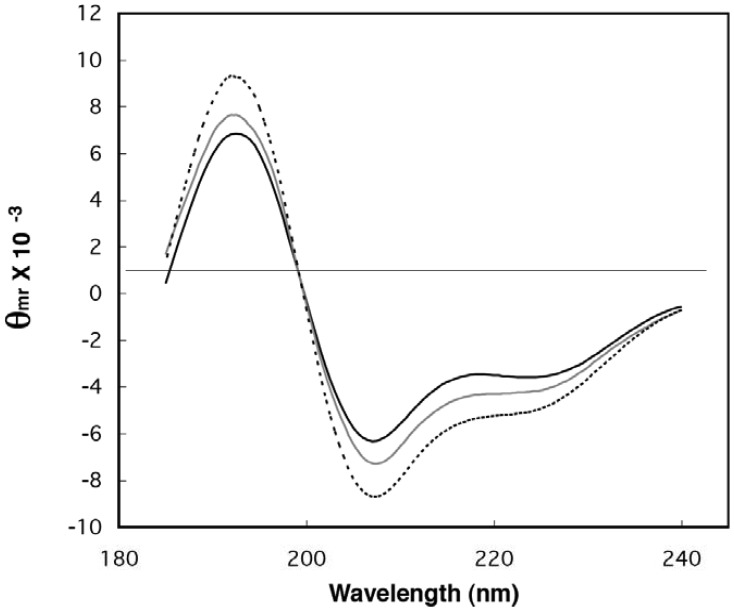
Circular dichroism (CD) spectra of ubiquitin and two libraries of random-sequence fusions. Spectra for ubiquitin (black line), UbLIB38 (gray line), and UbLIB71 (dotted line) represent averages over five or ten scans. Data are given in mean residue ellipticity.

The purified, novel fusion proteins (see Section 3.2 for purification details) were examined spectroscopically for signatures of folding commonly found in natural globular proteins. First, the secondary structure content of the pool samples was examined by circular dichroism (CD) spectroscopy (Figure 2). The characteristic α-helix peaks near 195, 208, and 222 nm are clearly increased for the library samples over those of ubiquitin, indicating helical regions within some of the random-sequence extensions. The CD spectra were deconvoluted using LINCOMB and the five basis spectra provided with the program [36]. The estimates of helix content match the conclusions drawn from visual inspection of the spectra. After subtracting the contribution due to ubiquitin from the library spectra, it was estimated that the random-sequences of LIB38 contained 23.6% α-helix and 20.5% β-sheet, while LIB71 contained 30.5% α-helix and 10.9% β-sheet. Interestingly, these estimates are within the range of secondary structure contents found in natural proteins [37]. The secondary structure values for the fusion libraries were calculated assuming a mean residue weight of 114 daltons and a mean length of 33.3 and 56.2 amino acids for the extensions in LIB38 and LIB71, respectively. Mean lengths were calculated using the expected termination codon frequency. LINCOMB deconvolution of control CD spectra (ubiquitin alone and as a mixture with myoglobin) gave helix and sheet estimates as expected based on known 3D structures [38].

**Figure 3:**
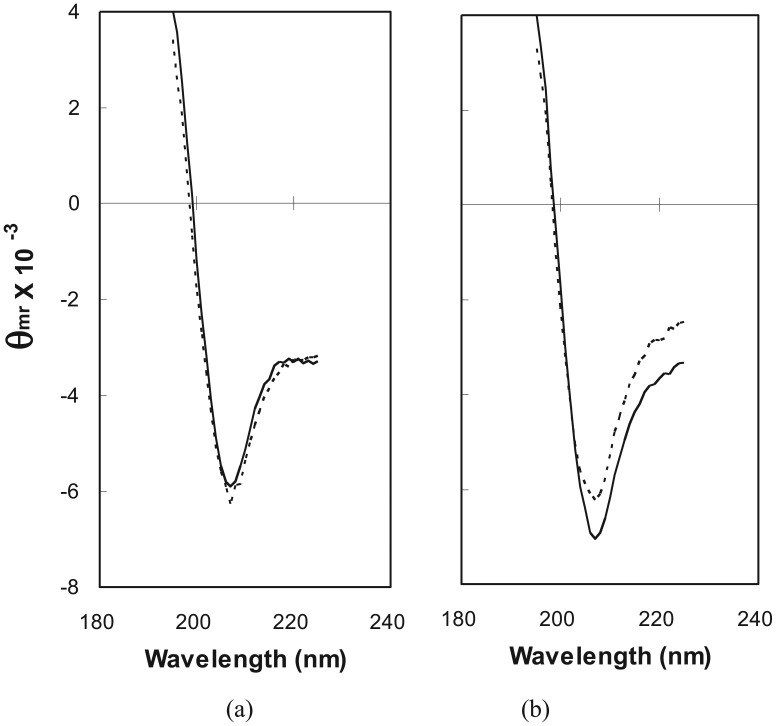
Effect of temperature on CD spectra of ubiquitin and fusion pool. CD spectra of (**a**) ubiquitin and (**b**) LIB71 are shown at 25 °C (solid line) and 75 °C (broken line). Data were collected as described in the Experimental Section, except using 10 repeat scans from 195 to 225 nm, in 10 °C increments with 10 minute equilibration time.

To further demonstrate absolute differences between the secondary structure content of control ubiquitin and a fusion pool sample, CD spectra were monitored during the heat-induced loss of secondary structure (Figure 3). Melting experiments demonstrated the reduction of helical content in LIB71 and stability of the ubiquitin spectrum during the change from 25 °C to 75 °C. Ubiquitin has been shown to denature at approximately 85 °C [39]. The decrease in ellipticity observed for LIB71 protein was essentially linear with increasing temperature, as might be expected for a complex mixture of proteins with distinct melting temperatures (data not shown). The loss of CD signal with increasing temperature clearly demonstrates, independent of possible artifacts from data normalization and deconvolution, the presence of heat-labile secondary structure in the random-sequence extensions.

### 2.2. Estimation of Folded Structure in Individual Random-Sequence Fusion Proteins

Individual fusions were chosen from the libraries at random with the stipulation that they contained DNA inserts of proper size as shown by restriction mapping. CD spectra were obtained for fifteen individual fusion proteins at 10 °C. Of the secondary structure estimates from CD data, helix estimates have been shown most reliable from controls here and previously [40], therefore, these helix values are used in further analyses. During processing of data on individual clones, normalization using polymer length was avoided since the lengths of individual sequences will deviate somewhat from the mean length of polymers in the library. Helix data from whole fusions are given in Table 1.

**Table 1:** Helix content estimated by deconvolution of circular dichroism (CD) spectra.

**Pool**	**Isolates**	**Helix content**	**Isolate averages** ^b^
**LIB38**		18.6*	

	38 mm	14.7*	16.6 ± 1.9
	38a	16.4	
	38c	15.7	
	38e	16.4	
	38j	16.2	
	38k	13.8*	
	38m	19.3*	
	38o	17.6*	
	38p	19.6*	

**LIB71**		22.4*	

	71c	19.5*	17.0 ± 1.8
	71d	18.2*	
	71e	14.4*	
	71h	17.0*	
	71j	15.9	
	71L	17.1*	

Ubiquitin			16.1 ± 0.04

a Data are given for whole fusions in order to avoid ubiquitin subtraction, since the exact length of individual novel extensions may deviate from the ensemble mean length.

b Means and standard deviation.

c * denotes greater than two standard deviations from the measured helix content in the ubiquitin control.

Next, individual protein fusions were probed for hydrophobic collapse by measuring their ability to protect intrinsic tryptophan side chains from solvent water. The maximum wavelength of tryptophan emission is determined by the polarity of its environment. With excitation at 280 nm, tryptophan fluoresces at or below ∼345 nm in a non-polar organic solvent or the interior of a protein, while in water the emission maximum is red shifted to ∼355 nm or above [41]. Thus, fluorescence emission (FE) measurement of the wavelength of maximum tryptophan fluorescence can be used as an indicator of structural collapse or compactness. Addition of sufficient denaturant to a native fold causes protein unfolding, exposure of protected tryptophan side chains, and a corresponding red-shift of the emission maximum. Ubiquitin contains no tryptophan and so its FE spectrum in the region of 345-355 nm is minimal and unaffected by denaturant. Any change observed in the FE spectra of the ubiquitin fusions upon addition of denaturant can therefore be attributed to alteration in the conformation of the random-sequence domain.

**Figure 4:**
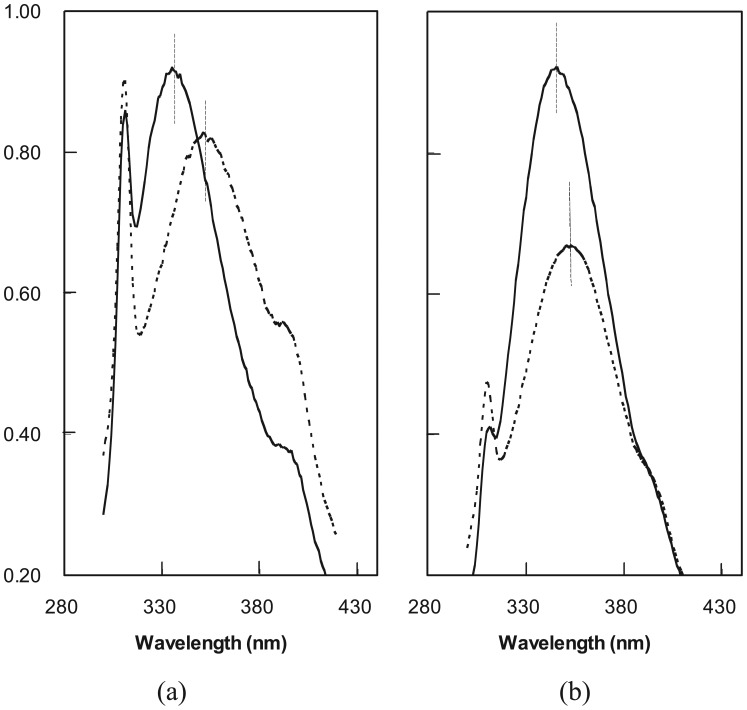
Fluorescence emission (FE) spectra of fusion protein pools (**a**) LIB38; and (**b**) LIB71. The spectra were taken in water (solid lines) and in 6 M GuHCl (broken lines). Decreased intensity and red shift of the emission wavelength maximum in GuHCl indicate protein unfolding and tryptophan exposure. The fluorescence emission of ubiquitin in this range was low and unaffected by GuHCl.

The two protein pools as well as 25 tryptophan-containing individual fusions were examined using FE spectroscopy under native and denaturing conditions. The spectra for LIB38 and LIB71 both show a red shift and decrease in intensity in the presence of 6 M GuHCl, consistent with protection of tryptophan from water in the absence of denaturant and exposure in its presence (Figure 4). This observation provides evidence of collapsed states in a large population of proteins in each fusion library. The change in emission intensity is also an indicator of structural alterations near tryptophan residues; however the direction and magnitude of such changes are unpredictable [41]. The reason for a larger red-shift and smaller intensity change in the LIB71 spectra versus that for LIB38 remains unclear. A large majority (19 out of 25) of the individual fusions examined also showed tryptophan protection. To test refolding of the random-sequence, reprotection of tryptophan was examined after performing buffer exchange from 6 M GuHCl back into Tris buffer. Reprotection was demonstrated for all six fusions tested (data not shown), thus collapsed structure is a property of the novel extensions, independent of *in vivo* protein biosynthesis.

The minimum structural requirements necessary for the protection of tryptophan from solvent have not been extensively investigated. However, using overlapping fragments of nicotinic acetylcholine receptor, it was observed that a 12-mer was incapable of protecting an intrinsic tryptophan but an 18-mer and a 32-mer were shown to collapse [42]. The present results suggest hydrophobic collapse is a rather common property of arbitrary, random-sequence polypeptides with amino acid compositions similar to biological proteins.

### 2.3. Cooperative Unfolding Shown by Intrinsic Fluorescence Emission

**Figure 5:**
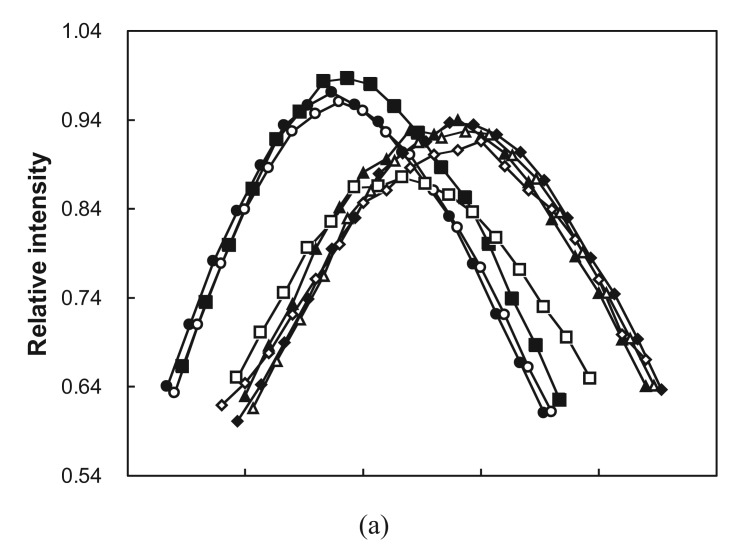

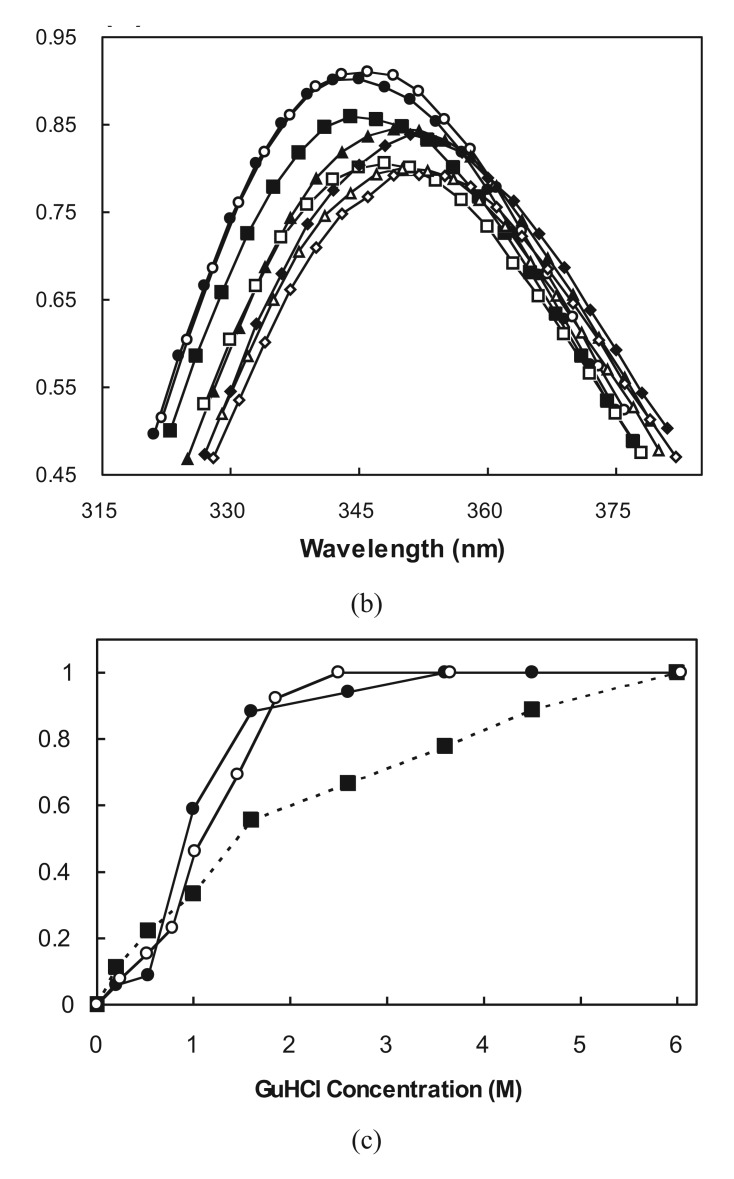
FE unfolding curves for two fusions with increasing GuHCl. For Ub71L **(a)** and Ub71h **(b)**. GuHCl concentrations: solid circles 0.0 M, open circles 0.2 M, solid squares 0.5 M, open squares 1 M, solid triangles 1.6 M, open triangles 2.6 M, solid diamonds 3.6 M, open diamonds 4.5 M. Spectra were also taken in 6 M GuHCl (not shown). **(c)** Fraction unfolded was calculated by dividing total observed red shift by the red shift at each denaturant concentration. Data are given for Ub71L attached to ubiquitin (solid circles) and cleaved from ubiquitin (open circles) and for Ub71h attached to ubiquitin (solid squares).

Having found evidence for helical structure and hydrophobic collapse, we next probed the cooperativity of the unfolding transitions in the random-sequence domains. The wavelength of maximum tryptophan fluorescence was monitored with increasing denaturant concentration to determine if loss of collapsed structure occurred gradually (non-cooperative) or rapidly (cooperative). Twelve fusions were denatured by incremental addition of GuHCl followed by equilibration and measurement of FE spectra. Two data sets and their unfolding profiles are shown (Figure 5). Three of the twelve fusions gave unfolding profiles similar to that for Ub71h (Figure 5b-c) in which the red shift of the FE peak occurred almost uniformly across the entire range of denaturant concentrations. This type of profile is indicative of non-cooperative unfolding. The majority of fusions (nine out of twelve) yielded unfolding profiles resembling that for Ub71L (Figure 5a-c) in which the red shift occurred non-uniformly. In these cases, a rapid increase in FE peak wavelength over a fairly small GuHCl concentration range indicated cooperative transitions. In all nine cases, the cooperative unfolding transitions occurred at GuHCl concentrations between 1 and 1.5 M which is within the low end of the range observed for unfolding of biological proteins [44]. This result does not distinguish between native and partially-folded structures, since cooperative unfolding has been observed previously in partially-folded designed proteins [43–45] and in molten globule states of natural proteins [46].

### 2.4. Ubiquitin Independent Unfolding and Refolding

One of the natural functions of C-terminal ubiquitin fusion is to minimize the degradation of poorly folding proteins during biosynthesis. Here, the influence of ubiquitin fusion on the folding of random sequence proteins was tested by cleaving the extensions from ubiquitin and examining their FE spectra with and without denaturant. UbCE hydrolase L1 was used to cleave ubiquitin fusions specifically between the C-terminal amino acid of ubiquitin and the N-terminal residue of the extensions. Eight proteins, which demonstrated tryptophan protection as ubiquitin fusions, were completely cleaved (as shown by SDS-PAGE), denatured with GuHCl, and refolded by buffer exchange. All eight of the clones displayed some degree of tryptophan protection in buffer and loss of protection in 6 M GuHCl. The unfolding of cleaved protein 71L was monitored with increasing GuHCl concentration. Its unfolding profile resembles that of the fusion protein, but appears slightly less cooperative (Figure 5(c)). These observations demonstrate that the folding of this arbitrary-sequence polypeptide is largely independent of attachment to ubiquitin.

### 2.5. Molten Globule-Like and Native-Like Behavior Suggested by ANS Binding Data

**Figure 6:**
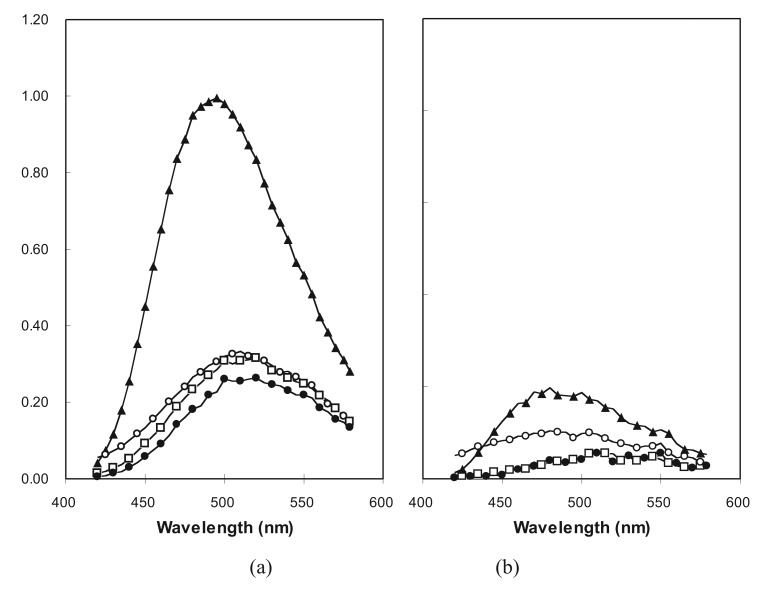
FE spectra of ANS with proteins: ubiquitin (solid circles), Ub38 mm (open circles), Ub71L (open squares), and Ub71h (solid triangles). Samples contained 10 μM protein and 250 μM ANS in 12.5 mM KH2PO4, 50 mM NaF, pH 7.5 containing either no GuHCl (**a**); or 2 M GuHCl (**b**). Excitation wavelength was 340 nm.

The fluorescent dye 1-anilino-naphthalene-8-sulfonate (ANS) has been shown previously to bind with higher affinity to the molten globule forms of natural proteins than to the native or unfolded states [47]. Presumably, this binding is due to the loose, and therefore accessible, hydrophobic core characteristic of the molten globule state. The intensity of emission by ANS is increased when the dye is sequestered from water within the interior of a protein, therefore binding is evident in FE spectra. Three fusion proteins were tested for association with ANS in buffer and in 2 M GuHCl (Figure 6). Ub38mm was chosen as a negative control, since it shows no evidence of a collapsed conformation, failing to protect an intrinsic tryptophan. Ub71h and Ub71L were examined because, while both protect tryptophan residues, the former unfolds non-cooperatively and the latter with marked cooperativity. While ubiquitin is stable in 2 M GuHCl [48], the two proteins from LIB71 are predominately unfolded at that concentration.

As expected, the intensity of ANS emission with Ub38mm was unaffected by denaturant indicating the lack of a collapsed conformation with an accessible hydrophobic core for the protein in buffer. Also, as expected, the spectrum of ANS with ubiquitin remains unchanged after addition of GuHCl, since ubiquitin is very stable and excludes ANS under both conditions. The intriguing result lies in the comparison of the spectra from the two LIB71 proteins. Ub71h caused a significant increase in the intensity of ANS FE in buffer (Figure 6(a)), but not in the presence of denaturant (Figure 6(b)). This indicates the existence of a denaturant-sensitive, collapsed conformation containing a hydrophobic core which is accessible to large organic molecules, analogous to the molten globule states of natural proteins. On the other hand, Ub71L caused no increase in the intensity of ANS fluorescence in buffer. Ub71L was shown to be larger than Ub71h (by SDS-PAGE) and to exist in a collapsed state (by intrinsic tryptophan FE), therefore it appears the packing in its core may be sufficiently tight to exclude ANS. By this measure, the behavior of Ub71L resembles that of a native protein more closely than that of a molten globule.

### 2.6. The Statistical Validity of a Random Sample of Molecular Sequences in vivo and in vitro

Since we are utilizing these protein libraries as random samples of unevolved proteins, we must consider spurious selective pressures applied during expression and purification. Since completely unfolded chains would be susceptible to degradation by host proteases, *in vivo* expression of polypeptides may automatically select for sequences with compact structure. In practice however, *in vivo* proteolysis was contraindicated by the fact that all thirty, randomly chosen, individual fusions examined were of greater apparent molecular weight than ubiquitin as shown by SDS-PAGE.

If proteolysis had been prevalent, one would have expected to see clones of unfused ubiquitin, since there is no reason to believe the encoded, stable ubiquitin would be degraded along with the susceptible extension. In addition, fusion with ubiquitin has been shown repeatedly to decrease proteolytic degradation of recombinant proteins [49] including a series of 10–21 residue peptides which are likely non-compact [35]. It is still possible that certain clones failed to grow at all. However, tight control of expression and brief induction followed by rapid purification limited the exposure of product to host cell contents and mitigated possible toxic effects on the cells.

Although selection is implicit in the process of protein purification, it is clear from the data presented here that polypeptides displaying ordered, folded conformations are easily obtained from pools that have not been stringently selected for specific functions. While there exist on the order of 10^46^ and 10^86^ possible sequences consistent with libraries LIB38 and LIB71, respectively, we have examined only a small number (approximately 30 individual proteins). Such profoundly sparse sampling would render a lack of evidence for structure uninterpretable, since failure to observe some property would not disprove its existence. On the other hand, positive evidence of structure, such as our observations of collapsed conformations, cooperative unfolding, and secondary structure, conclusively demonstrate that these properties are surprisingly common in sequence space.

### 2.7. Rudimentary Folds

Within the compositional régime of protein sequence space examined here, it is evident that sequences having collapsed conformations with cooperative folding transitions and measurable levels of secondary structure are common and easily discovered by a random search. These structural properties are not unlike those observed among induced molten globule states of perturbed native folds [50]. To differentiate the two, we refer to the observed, partially ordered conformational states of unevolved sequences as *rudimentary folds*. Since rudimentary folding seems to resemble a molten globule state more closely than an unfolded state, native folds are, most likely, also more common than previously suspected. Indeed, in our sparse sampling, we have discovered at least one example protein (71L) exhibiting native-like folding characteristics.

These results are similar to and consistent with those of computational and laboratory experiments comparing the folded structures of random-sequence single-stranded RNAs [51–53]. For example, *in silico* folding of randomized RNA sequences demonstrates that unevolved RNAs have the same structure-dependent compositional biases as those observed for rRNAs [54–56]. In laboratory experiments using a battery of complementary structural probes (in this case, native gel electrophoresis, analytical centrifugation and lead-II hydrolysis), it was found that unevolved RNAs (having sequence length and nucleotide composition analogous to evolved sequences) adopted sequence-specific, magnesium-dependent folding to states as compact as those documented for cognate biological RNAs [57]. Yet, the authors conclude that the secondary structural elements of these unevolved RNAs failed to attain the unique tertiary contacts that characterize native RNA folds. This observation that typical random-sequence RNA adopts a relatively small number of compact states is directly analogous to the molten globule-like states described herein for random-sequence proteins. Hence, rudimentary folding appears to be a common feature of both RNA and protein polymeric systems.

## 3. Experimental Section

### 3.1. Library Construction

A detailed description of the library design and construction is published elsewhere [30,34]. Briefly, the cloning procedure (summarized in Figure 1) utilized synthetic oligonucleotides each containing a variable region flanked on each end by constant primer-binding sequences. The randomized codons were constructed by adding premixed nucleoside phosphoramidites during the elongation step of standard oligonucleotide synthesis. Thus, the exact sequence of any individual molecule was determined partially by chance, while the ensemble average was dictated by the input nucleotide ratios. The nucleotide mixtures were designed to bias the probabilities away from termination codons and toward an amino acid composition similar to the mean for natural, globular proteins [30]. In the present libraries, the input molar ratios for T:C:A:G (in percentage) were 8:21:32:39 in the first codon position, 24:25:28:23 in the second position, and 30:30:0:40 in the third. The reactivities of the four nucleoside phosphoramidites were shown previously to be essentially equal in cases where fresh, anhydrous solutions were used [58–60].

The constant regions of the synthetic DNA contained, on the 3′ end, a Bam HI restriction site and on the 5′ end, a Bgl II site. These enzymes generate compatible overhangs and were used to ensure proper orientation of the fragments in the plasmid while allowing for stepwise addition of an unlimited number of inserts. The actual length of any individual fusion protein is determined not only by the number of inserted fragments but also by chance, since the probability of encoding a termination codon is less than 0.007 per codon in these libraries. Protein samples were produced and purified for two fusion libraries, LIB38 (30,000 clones) and LIB71 (19,000 clones), having one or two inserts, respectively and for unmodified ubiquitin (produced from pNMHUB, identical to pNMHUBpoly except the poly-linker following the ubiquitin gene is replaced by an in-frame termination codon). Expression and purification of the fusions proteins is outlined in the methods section below. Proteins were shown to be homogeneous by Coomassie blue stained, overloaded SDS-PAGE. Experimental analysis of the overall amino acid compositions of the fusion protein pools was described previously and indicated a rich, well-balanced amino acid composition coincident with the biological region of sequence we targeted for investigation [34].

### 3.2. Protein Purification

Plasmid libraries were transformed into hexamminecobalt chloride treated *E. coli* AR68 [61], a protease deficient strain which constitutively expresses a heat labile λ-phage cI repressor protein [62]. Transformants were grown on LB plates containing 75 μg/mL ampicillin, counted to establish library diversity, and suspended in LB medium. Individuals and pools of clones were grown at 30 °C then heat shocked at 42 °C to induce production of the fusions. Cells were lysed by sonication and the supernatant from a high-speed centrifugation was passed over a Q-Sepharose FPLC column (Pharmacia LKB) in 20 mM Tris/HCl, pH 7.5, 50 mM NaCl, 0.03% sodium azide. Ubiquitin and the ubiquitin fusions passed through the column without binding; peak fractions were collected, concentrated by ultrafiltration, and passed over a Sephadex G-50 FPLC column. This gel permeation step, in most cases, yielded a peak of fusion protein shown to be pure by polyacrylamide electrophoresis. If necessary, peak fractions were concentrated and the G-50 step repeated.

### 3.3. Fusion Protein Hydrolysis

The fusion Ub71L was digested with UbCE hydrolase L1 [63] at 37 °C in 20 mM DTT, 20 mM TrisHCl, pH 7.5, 50 mM NaCl for 40 hours, then denatured in 5 M GuHCl, and refolded into 12.5 mM KH_2_PO_4_, 50 mM NaF, pH 7.5 by buffer exchange using a Centra-Con 3 ultrafiltration unit (Amicon). Cleavage of the novel carboxy extension protein from ubiquitin was shown to be complete by SDS-PAGE.

### 3.4. Spectroscopy

CD spectra were taken on an Aviv 62DS spectropolarimeter. Five or ten repeat scans were collected per sample with 0.5 nm step size and 2 second averaging time. Proteins (10–25 μM) were in 12.5 mM KH_2_PO_4_, 50 mM NaF, pH 7.5, at 25 °C in either a 1 or 2 mm path cuvette. The normalization and deconvolution of CD spectra is critically dependent upon the protein concentration values used in normalizing the curves, therefore, samples were analyzed in triplicate using the micro-BCA assay (Pierce, Rockford, IL). Protein concentrations measured by BCA assay were verified by comparison with quantitative amino acid analysis results for several samples.

FE spectra were obtained using an SLM Spectrofluorimeter. A rectangular micro-cuvette was used with 130 μL protein sample (5–10 μM) in water and in 6 M GuHCl. Data were collected from 300 to 420 nm with 1 nm step size, 280 nm excitation wavelength. In the FE monitored unfolding experiments aliquots of 8 M GuHCl were added manually to the cuvette, mixed by pipette trituration, and equilibrated for 1–2 minutes before collecting the spectra. Spectra were normalized by baseline subtraction and dilution correction. Samples in the ANS binding experiments contained 10 μM protein and 250 μM ANS in 12.5 mM KH_2_PO_4_, 50 mM NaF, pH 7.5 containing either no GuHCl or 2 M GuHCl. Data were collected over the range 420 to 580 nm with 340 nm excitation.

## 4. Conclusions

In this study, we have created combinatorial protein pools with amino acid compositions near those observed in globular proteins found in nature [30]. An intriguing future direction would be to synthesize combinatorial pools having amino acid compositions similar to other classes of protein structures (such as fibrous [64], membrane [65], and the more recently discovered intrinsically unstructured [66] proteins). Might random protein sequences having these distinct amino acid compositions have correspondingly distinct folding properties? Ultimately, it will be necessary to explore the vast compositional régimes of sequence space that are not occupied by biological sequences. Would these regions display lower (or possibly higher) frequencies of native folding? Of rudimentary folding?

It has been assumed that biological proteins fold into specific three-dimensional conformations because evolution has acted over billions of years to select those rare sequences having this capability. The evidence presented herein indicates that synthetic proteins having unevolved sequences possess many features of folding usually considered to be derived evolutionary adaptations. Although selection is essential for the evolution of specific, functional protein folds, it is not required to account for secondary structure and compact, cooperative folding.

## References

[1] Van Holde K.E. (2003). Reflections on a century of protein chemistry. Biophys. Chem..

[2] Frauenfelder H., Wolynes P.G. (1994). Biomolecules: Where the physics of complexity and simplicity meet. Phys. Today.

[3] Meier S., Özbek S. (2007). A Biological cosmos of parallel universes: Does protein structural plasticity facilitate evolution?. BioEssays.

[4] Kimura M. (1968). Evolutionary rate at the molecular level. Nature.

[5] King J.L., Jukes T.H. (1969). Non-Darwinian evolution. Science.

[6] Salisbury F.B. (1969). Natural selection and the complexity of the gene. Nature.

[7] Smith J.M. (1970). Natural Selection and the Concept of Protein Space. Nature.

[8] Keefe A.D., Szostak J.W. (2001). Functional proteins from a random-sequence library. Nature.

[9] Meyerguz L., Kleinberg J., Elber R. (2007). The network of sequence flow between protein structures. Proc. Natl. Acad. Sci. USA.

[10] Prymula K., Piwowar M., Kochanczyk M., Flis L., Malawski M., Szepieniec T., Evangelista G., Minervini G., Polticelli F., Wiśniowski Z. (2009). *In silico* structural study of random amino acid sequence proteins not present in nature. Chem. Biodiv..

[11] Minervini G., Evangelista G., Villanova L., Slanzi D., de Lucrezia D., Poli I., Luisi P.L., Polticelli F. (2009). Massive non-natural proteins structure prediction using grid technologies. BMC Bioinf..

[12] Cunningham B.C., Wells J.A. (1989). High-resolution epitope mapping of hGH-receptor interactions by alanine-scanning mutagenesis. Science.

[13] Hermes J.D., Blacklow S.C., Knowles J.R. (1990). Searching sequence space by definably random mutagenesis: Improving the catalytic potency of an enzyme. Proc. Natl. Acad. Sci. USA.

[14] Lim W.A., Sauer R.T. (1991). The role of internal packing interactions in determining the structure and stability of a protein. J. Mol. Biol..

[15] Hellinga H.W., Wynn R., Richards F.M. (1992). The hydrophobic core of Escherichia coli thioredoxin shows a high tolerance to nonconservative single amino acid substitutions. Biochemistry.

[16] Sondek J., Shortle D. (1990). Accommodation of single amino acid insertions by the native state of staphylococcal nuclease. Proteins.

[17] Urfer R., Kirschner K. (1992). The importance of surface loops for stabilizing an eightfold beta alpha barrel protein. Protein Sci..

[18] Bloom J.D., Raval A., Wilke C.O. (2007). Thermodynamics of neutral protein evolution. Genetics.

[19] Yeates T.O. (2007). Protein structure: Evolutionary bridges to new folds. Curr. Biol..

[20] Schultes E., Bartel D.P. (2000). One sequence, two ribozymes: Implications for the emergence of new ribozyme folds. Science.

[21] Xia Y., Levitt M. (2004). Simulating protein evolution in sequence space and structure space. Curr. Opin. Struct. Biol..

[22] Katchelski E., Sela M. (1958). Synthesis and chemical properties of poly-alpha-amino acids. Adv. Prot. Chem..

[23] Rao S.P., Carlstrom D.E., Miller W.G. (1974). Collapsed structure polymers: A scattergun approach to amino acid copolymers. Biochemistry.

[24] Anufrieva E.V., Bychkova V.E., Krakovyak M.G., Pautov V.D., Ptitsyn O.B. (1975). A synthetic polypeptide with a compact structure and its self-organization. FEBS Lett..

[25] Davidson A.R., Sauer R.T. (1994). Folded Proteins Occur Frequently in Libraries of Random Amino Acid Sequences. Proc. Natl. Acad. Sci. USA.

[26] Davidson A.R., Lumb K.J., Sauer R.T. (1995). Cooperatively folded proteins in random sequence libraries. Nat. Struct. Biol..

[27] Doi N., Kakukawa K., Oishi Y., Yanagawa H. (2005). High solubility of random-sequence proteins consisting of five kinds of primitive amino acids. Prot. Eng. Des. Sel..

[28] Prijambada I.D., Yomo T., Tanaka F., Kawama T., Yamamoto K., Hasegawa A., Shima Y., Negoro S., Urabe I. (1996). Solubility of artificial proteins with random sequences. FEBS Lett..

[29] Chiarabelli C., Vrijbloed J.W., de Lucrezia D., Thomas R.M., Stano P., Polticelli F., Ottone T., Papa E., Luisi P.L. (2006). Investigation of de novo totally random biosequences. Part II. On the folding frequency in a totally random library of de novo proteins obtained by phage display. Chem. Biodiv..

[30] LaBean T.H., Kauffman S.A. (1993). Design of synthetic gene libraries encoding random sequence proteins with desired ensemble characteristics. Prot. Sci..

[31] Monia B.P., Ecker D.J., Jonnalagadda S., Marsh J., Gotlib J.L., Butt T.R. (1989). Gene synthesis, expression and processing of human ubiquitin carboxy extension proteins in bacteria and yeast. J. Biol. Chem..

[32] Butt T.R., Jonnalagadda S., Monia B.P., Sternberg E.J., Marsh J.A., Stadel J.M., Ecker D.J., Crooke S.T. (1989). Ubiquitin fusion augments the yield of cloned gene products in Escherichia coli. Proc. Natl. Acad. Sci. USA.

[33] Yoo Y., Rote K., Rechsteiner M. (1989). Synthesis of peptides as cloned ubiquitin extensions. J. Biol. Chem..

[34] LaBean T.H., Kauffman S.A., Butt T.R. (1995). Libraries of random-sequence polypeptides produced with high yield as carboxy-terminal fusions with ubiquitin. Mol. Div..

[35] Wilkinson K.D., Lee K.M., Deshpande S., Duerksen-Hughes P., Boss J.M., Pohl J. (1989). The neuron-specific protein PGP 9.5 is a ubiquitin carboxyl-terminal hydrolase. Science.

[36] Perczel A., Hollosi M., Tusnady G., Fasman G.D. (1991). Convex constraint analysis: A natural deconvolution of circular dichroism curves of proteins. Prot. Eng..

[37] Kabsch W., Sander C. (1983). Dictonary of protein secondary structure: Pattern recognition of hydrogen-bonded and geometrical features. Biopolymers.

[38] Jenson J., Goldstein G., Breslow E. (1980). Physical-chemical properties of ubiquitin. Biochim. Biophys. Acta.

[39] Cary P.D., King D.S., Crane-Robinson C., Bradbury E.M., Rabbani A., Goodwin G.H. (1980). Structural studies on two high-mobility-group proteins from calf thymus, HMG-14 and HMG-20 (ubiquitin), and their interaction with DNA. Eur. J. Biochem..

[40] Johnson W.C.J. (1988). Secondary structure of proteins through circular dichroism spectroscopy. Annu. Rev. Biophys. Biophys. Chem.

[41] Lakowicz J.R. (1983). Principles of Fluorescence Spectroscopy.

[42] Pearce S.F., Hawrot E. (1990). Intrinsic fluorescence of binding-site fragments of the nicotinic acetylcholine receptor: Perturbations produced upon binding alpha-bungarotoxin. Biochemistry.

[43] Saito Y., Wada A. (1983). Comparative study of GuHCl denaturation of globular proteins. II. A phenomenological classification of denaturation profiles of 17 proteins. Biopolymers.

[44] Hecht M.H., Richardson J.S., Richardson D.C., Ogden R.C. (1990). De novo design, expression, and characterization of Felix: A four-helix bundle protein of native-like sequence. Science.

[45] Regan L., DeGrado W.F. (1988). Characterization of a helical protein designed from first principles. Science.

[46] Uversky V.N., Semisotnov G.V., Pain R.H., Ptitsyn O.B. (1992). ‘All-or-none’ mechanism of the molten globule unfolding. FEBS Lett..

[47] Semisotnov G.V., Rodionova N.A., Razgulyaev O.I., Uversky V.N., Gripas A.F., Gilmanshin R.I. (1991). Study of the “molten globule” intermediate state in protein folding by a hydrophobic fluorescent probe. Biopolymers.

[48] Lenkinski R.E., Chen D.M., Glickson J.D., Goldstein G. (1977). Nuclear magnetic resonance studies of the denaturation of ubiquitin. Biochim. Biophys. Acta.

[49] Butt T.R., Jonnalagadda S., Monia B.P., Sternberg E.J., Marsh J.A., Stadel J.M. (1989). Ubiquitin fusion augments the yield of cloned gene products in. Escherichia coli. Proc. Natl. Acad. Sci. USA.

[50] Ptitsyn O.B. (1995). Molten globule and protein folding. Adv. Prot. Chem..

[51] Schultes E., Hraber P.T., LaBean T.H. (1997). Global similarities in nucleotide base composition among disparate functional classes of single-stranded RNA imply adaptive evolutionary convergence. RNA.

[52] Schultes E., Hraber P.T., LaBean T.H. (1999). A parameterization of RNA sequence space. Complexity.

[53] Schultes E., Hraber P.T., LaBean T.H. (1999). Estimating the contributions of selection and self-organization in RNA secondary structures. J. Mol. Evol..

[54] Smit S., Yarus M., Knight R. (2006). Natural selection is not required to explain universal compositional patterns in rRNA secondary structure categories. RNA.

[55] Smit S., Yarus M., Knight R. (2009). RNA structure prediction from evolutionary patterns of nucleotide composition. Nucl. Acids Res..

[56] Kennedy R., Lladser M.E., Wu Z., Zhang C., Yarus M., de Sterck H., Knight R. (2010). Natural and artificial RNAs occupy the same restricted region of sequence space. RNA.

[57] Schultes E.A., Spasic A., Mohanty U., Bartel D.P. (2005). Compact and ordered collapse in randomly generated RNA sequences. Nat. Struct. Mol. Bio..

[58] Bartel D.P., Szostak J.W. (1993). Isolation of new ribozymes from a large pool of random sequences. Science.

[59] Zon G., Gallo K.A., Samson C.J., Shao K.L., Summers M.F., Byrd R.A. (1985). Analytical studies of ‘mixed sequence’ oligodeoxyribonucleotides synthesized by competitive coupling of either methyl- or beta-cyanoethyl-N,N-diisopropylamino phosphoramidite reagents, including 2′-deoxyinosine. Nucl. Acids Res..

[60] Mandecki W.A. (1990). A method for construction of long randomized open reading frames and polypeptides. Prot. Eng..

[61] Sambrook J., Fritsch E.F., Maniatis T. (1989). Molecular Cloning: A Laboratory Manual.

[62] Mott J.E., Grant R.A., Ho Y.S., Platt T. (1985). Maximizing gene expression from plasmid vectors containing the lambda PL promoter: Strategies for overproducing transcription termination factor rho. Proc. Natl. Acad. Sci. USA.

[63] Wilkinson K.D., Cox M.J., Mayer A.N., Frey T. (1986). Synthesis and characterization of ubiquitin ethyl ester, a new substrate for ubiquitin carboxyl–terminal hydrolase. Biochemistry.

[64] Nadiger G.S., Bhat N.V., Padhye M.R. (1985). Investigation of amino acid composition in the crystalline region of silk fibroin. J. App. Pol. Sci..

[65] Tusnády G.E., Simon I. (1998). Principles governing amino acid composition of integral membrane proteins: Application to topology prediction. J. Mol. Bio..

[66] Tompa P. (2002). Intrinsically unstructured proteins. Trends Biochem. Sci..

